# Trends in patient satisfaction in Dutch university medical centers: room for improvement for all

**DOI:** 10.1186/s12913-015-0766-7

**Published:** 2015-03-19

**Authors:** Sophia M Kleefstra, Linda C Zandbelt, Hanneke JCJM de Haes, Rudolf B Kool

**Affiliations:** Department Research and Innovation, Dutch Health Care Inspectorate, Utrecht, the Netherlands; Academic Medical Center, department Quality and Process Innovation, University of Amsterdam, Amsterdam, the Netherlands; Academic Medical Center, department Medical Psychology, University of Amsterdam, Amsterdam, the Netherlands; Radboud Institute for Health Sciences, IQ healthcare, Radboud University Medical Center, Nijmegen, the Netherlands

**Keywords:** Patient satisfaction, Trend study, Hospitals, Quality improvement, Patient-centered care

## Abstract

**Background:**

Results of patient satisfaction research provide hospitals areas for quality improvement. Although it may take several years to achieve such improvement, not all hospitals analyze changes in patient satisfaction over time structurally. Consequently, they lack information from patients’ perspective on effectiveness of improvement programs. This study presents a trend analysis of the patient satisfaction scores in the eight university medical centers in the Netherlands. We focus on the trends, effect size and its consequences for improving patient-centered care.

**Methods:**

The Core Questionnaire for the assessment of Patient satisfaction (COPS) was used in four large-scale nationwide comparative studies (2003–2009). Data were analyzed at a national level, and for each academic hospital separately. We analyzed the polynomial contrasts in the four measurements by performing an univariate analysis of variance (ANCOVA). The trend lines are presented graphically, with the means, SD, F-statistics and the standardized effect size including confidence intervals expressed by Cohen’s d. By analyzing the (logit transformed) percentages of very satisfied patients we examined the change scores.

**Results:**

The dataset consisted of 58,055 inpatients and 79,498 outpatients. Significant positive trends were found on national level and hospital level, especially in outpatient departments. Improvement was especially seen on the dimensions “information” and “discharge and aftercare”. Not only university medical centers with a lower score at the start, but surprisingly some best practices and university medical centers with a high initial score improved.

**Conclusions:**

We conclude that significant trends in patient satisfaction can be identified on a national and a hospital level, in inpatient and outpatient departments. The observed effect size expressed by Cohen’s d is rather small. Hospitals have found room for improvement, even hospitals with initial high satisfaction scores. We recommend that hospitals monitor their patient satisfaction scores over time and relate these to quality interventions and organizational changes. Furthermore, we recommend to expand the research to subgroups of unsatisfied patients to improve patient-centered care for all patients.

**Electronic supplementary material:**

The online version of this article (doi:10.1186/s12913-015-0766-7) contains supplementary material, which is available to authorized users.

## Background

Over the last decades hospitals have been working on improving patient-centered care by developing and implementing quality improvement strategies and activities based on the patients’ perspective [1-6]. The Institute of Medicine defines patient-centered care as: “Providing care that is respectful of and responsive to individual patient preferences, needs, and values, and ensuring that patient values guide all clinical decisions” [7]. Results of patient experience and satisfaction research can inform hospitals of areas requiring improvement from patients’ perspective [2,3,5,6,8]. In fact, the continuous assessment of patients’ perspective is increasingly recognized as a major component of quality management [9]. Several studies have shown that significant improvement in most aspects of patient experience or satisfaction can be achieved over time [2,5,6,8-12], provided that organizations have adopted a strategic organizational approach to patient focus [11,13]. This cultural change will probably take several years to be implemented. Consequently, it takes time to achieve improvement in patient satisfaction or experiences [3,11].

Therefore, by analyzing patient satisfaction scores over time, hospitals can monitor whether their quality interventions result in better outcomes and assess hereby the effectiveness of improvement programs from the perspective of the patient [2-6,11-13]. In practice, however, not all hospitals analyze changes in patient satisfaction scores over time structurally [3,4,6]. As a consequence, these hospitals lack information on effectiveness of their programs for improving patient-centered care.

It is therefore no surprise that only a few studies were performed concerning trends over time in patient satisfaction on a national or an organizational level. There is also hardly any research that links trends to improvements hospitals made based on previous patient satisfaction research. For instance, the NHS national surveys show significant improvement in most aspects of patients’ experiences of inpatient and outpatient care and treatment in the UK (NHS, 2003–2009) [2,5]. In South Korea, patient satisfaction increased dramatically in inpatient and outpatient care (1989–2003), due to governmental policies on increased health expenditures, better availability of resources and quality improvement efforts [10]. In Denmark, patient satisfaction results on a national level for outpatients, day care surgical patients and medical patients improved, but remained unchanged for inpatients (1999–2006). Changes in patient satisfaction showed a so called ceiling effect: the best scoring departments had little or no room for improvement in patient satisfaction [6].

Patient surveys are generally accepted tools to monitor quality performance from the perspective of the patient, provided that the results should be attributed to smaller units than organizational level, as well as combined with qualitative and organizational data [4-6,11]. At the same time, there are some objections to the use of continuous patient satisfaction surveys for quality improvement. First, some consider patient satisfaction as a subjective judgment, and difficult to interpret [2,14]. Second, in general patients tend to be highly satisfied with their hospital care and these high scores are said to be difficult to improve. As a consequence, patient satisfaction questionnaires often show a skewed score distribution [9,15-17]. Activities highly successful in improving other indicators can increase the already high mean satisfaction scores by only a limited extent [14,18].

Indeed, research regarding trends in patient satisfaction shows that the biggest improvement takes place in departments with originally the lowest patient satisfaction and that departments with high scores hardly can improve theirs [6,19]. Such ceiling effect would imply that repeated measurements tend to lose their impact over time. Once an acceptable score for hospital standards has been achieved it would become difficult to develop successful initiatives that lead to further improvement [6]. This would be a serious obstacle to improving patient centered healthcare based on patient satisfaction scores.

In this study, we investigate patient satisfaction data from the eight university medical centers in the Netherlands. They performed large scale nationwide comparative studies with the same instrument and the same procedure two-yearly from 2003 [20]. The added value of these as compared to earlier studies is that feedback was detailed on a low organizational level i.e., inpatient and outpatient departments, with next to the quantitative qualitative data collected by asking patients’ free-text comments. Based on these measurements, the university medical centers made quality interventions such as redesigning the patient flow to reduce waiting times and increase accessibility, distributing patient leaflets, creating websites for patients and organizing courses in hospitality for staff. This cycle of measurement and improvement was repeated with the same methods three times until 2009.

Using the data of these four measurements, we addressed the following questions. (1) Can significant trends in patient satisfaction be identified on a national and a hospital level, in inpatient and outpatient departments? (2) Do hospitals with initial high satisfaction scores find room for improvement? By answering these questions, we hope to contribute to the discussion whether patient surveys over time offer structural opportunities for monitoring and improving patient-centered care.

## Methods

In a time series design we analyzed patient satisfaction data collected from all eight university medical centers in the Netherlands [20]. These data covered the measurements for 2003, 2005, 2007 and 2009.

### Instrument

Patient satisfaction was assessed using the Core Questionnaire for the assessment of Patient Satisfaction (COPS). The COPS is a reliable and valid Dutch questionnaire in a clinical and outpatient version. Further information on the development and validation the COPS questionnaire can be found in an earlier publication [20]. The COPS consists of six quality dimensions: “Admission”/“Reception” (2 or 3 items), “Nursing care” (2 items), “Medical care” (2 items), “Information” (4 items), “Autonomy” (2 or 3 items) and “Discharge and aftercare” (2 or 3 items). The six dimensions of the inpatient and outpatient version are equal, however some questions are different, given the different nature of the inpatient and outpatient departments. For three dimensions the content and number of items thus slightly varied. Patients can rate their satisfaction on a 5-point Likert-scale with answering categories unsatisfied (=1), somewhat satisfied (=2), rather satisfied (=3), quite satisfied (=4) and very satisfied (=5). The labels were spaced as comparably as possible from a semantic point of view. An intentionally skewed wording of answering categories was chosen, i.e. with one label “negative” and four labels “positive”, as patients are likely to give answers to positively framed responses rather than to negative ones [9,21]. Dimension scores were composed by adding up the item scores and dividing the resulting total score by the number of items. To establish the overall satisfaction we calculated the mean of the six dimensions of the COPS. Patients could write down comments using free text space after every dimension and at the end of the questionnaire. These comments were used by the hospitals to make quality improvements, but they were not part of the analyses in this trend study. The questionnaire included patients’ background characteristics such as age, gender and education. Finally, patients were asked to rate their general health on a 5-point scale (1 = bad, 2 = moderate, 3 = good, 4 = very good, 5 = excellent).

### Sample and procedure

The COPS was used in four large-scale nationwide comparative studies in all eight university medical centers in the Netherlands. These university medical centers are geographically spread throughout the Netherlands. The capital Amsterdam has two centers. In 2011 the eight hospitals had on average 948 beds (range 715–1320), on average 6887 full-time equivalent professionals (range 4113–9674), on average 31,430 admissions (range 21,161-41,797) comprehending on average 204,347 days (range 125,811-288,799), on average 138,260 first outpatient visits (range 123,435-157,665) an average length of stay of 6.7 days (range 5.6-8.7). The main tasks are complex patient care, experimental research and education [22,23].

The study sample was stratified according to the main medical specialties, i.e. 17 clinical and 27 outpatient specialties. Based on pilot studies [20,21,24] two hundred fifty consecutive patients were approached from every medical specialty in each hospital. We did not perform a power analysis to confirm the preferred power level. A coordinator was appointed in each university medical center and instructed to ensure a comparable approach across the eight hospitals.

In 2003 and 2005, COPS was sent to patients within two months after admission or an outpatient visit, accompanied by a letter from the hospital. Specific information was given in the letter in English, French, German, Spanish, Turkish and Moroccan, inviting patients to ask the help of others in case they were unable to read Dutch. Questionnaires could be returned to an independent research organization in a pre-stamped return envelope. A reminder was sent after 2 weeks.

In 2007 and 2009, the same procedure was followed, supplemented by the possibility to complete the questionnaire online using a personal code in the letter. It remained possible to send the questionnaire back by mail. During all four measurement periods a helpdesk using phone and email was installed for patients needing support. Prior to each measurement round the university medical centers reported the quality interventions they made based on the previous measurement to the independent research organization.

### Analyses

To answer our first research question, we examined the presence of significant trend lines on a national and a hospital level [25], for inpatient and outpatients departments. We analyzed the polynomial contrasts in the four measurements by performing a univariate analysis of variance (ANCOVA). Patients’ age, education and health status, which are known to influence patient satisfaction, were significantly different across the four measurements. Therefore, these were taken as a covariate in the analyses. As multilevel analyses on this data in an earlier study [26] revealed that differences in satisfaction scores were mainly determined at the patient level and to a very little extent at the department and the hospital level (ICC’s between 0 and 0.04), it was considered unnecessary to account for the hierarchical structure of the data. The confidence intervals were adjusted for multiple comparisons by Bonferroni. Subsequently, we determined the effect of the trend line. The effect of a polynomial contrast is determined by the number of peaks and falls in the curve. The following contrasts are possible:

- linear, i.e., a line that has no peak or fall and can be increasing or decreasing,
- quadratic, i.e., a curve with a peak or fall and,
- cubic, i.e., a curve with two bending points.

If there is more than one significant trend line, the smallest significance level indicates the dominant trend line. If the significance of both trend lines is close, the plot indicates the trend line. We only report significant trend lines. The trend lines are presented graphically, with the means, SD, F-statistics and the standardized effect size including confidence intervals expressed by Cohen’s d. Cohen’s d is an accepted measure of the standardized effect size. Using Cohen’s d facilitates interpretation of change since it can be compared to standards of size and a large amount of literature [27]. We related the interpretation of the effect size to Cohen’s conventions [28,29], in absence of effect sizes (/Cohen’s d) in previous patient satisfaction publications: a standardized effect size of 0.2 is considered “small” in magnitude, an effect size of 0.5 “medium” and an effect size of 0.8 “large”.

To answer our second research question, we first checked our data for two possible ceiling effects. First, the score limitation at the top of a scale [9,30] of the questionnaire, i.e. the percentage of patients giving the highest possible score (namely 5 = very satisfied). The COPS was designed with an intentionally skewed wording of answering categories to prevent such ceiling effect. In clinical research (health status questionnaires) a ceiling effect of 15 per cent is considered the maximum acceptable [31,32]. We calculated the overall percentages very satisfied patients by adding the ‘“very satisfied”’-scores per item, divided by the number of items in the questionnaire. Furthermore, we computed the absolute increase of percentage very satisfied patients between 2003 and 2009 to indicate change. We logit transformed the percentage scores to account for the potential numerical ceiling effect, i.e. the fact that it is more difficult to achieve a change from 90 to 95% than it is from 60 to 65% [33]. Data were analyzed using IBM SPSS 20.0.

## Results

### Sample

The total dataset of the inpatient departments consisted of 58,055 patients, the dataset of outpatients of 79,498 patients (see Additional file 1: Table S1). Patients visited the 17 (clinic) and 22 (outpatient) main medical specialties in the Netherlands. The response rates of 2003, 2005 and 2007 were consistently 53 percent. In 2009 the response rate was 42 percent. The patients’ level of education, age and health status differed significantly between the four measurements. In 2009 both the inpatient and outpatient respondents were older, higher educated and assessed their health status as less negative compared to the earlier measurements (see Additional file 1: Table S2 and S3).

### Are significant trends in patient satisfaction found on a national and a hospital level, in inpatient and outpatient departments?

The overall patient satisfaction scores on a national level showed a significant linear increase in the time period 2003 – 2009 for inpatient and outpatient departments. For the inpatients the mean satisfaction increased 0.05 on a 5 point scale: F (df) 3.857 (3), Cohen’s d 0.07 (95% CI 0.04-0.10), p = 0.009; for outpatients 0.09 F (df) 18.468 (3), Cohen’s d 0.12 (95% CI 0.09-0.15), p < 0.001.

**Figure 1:**
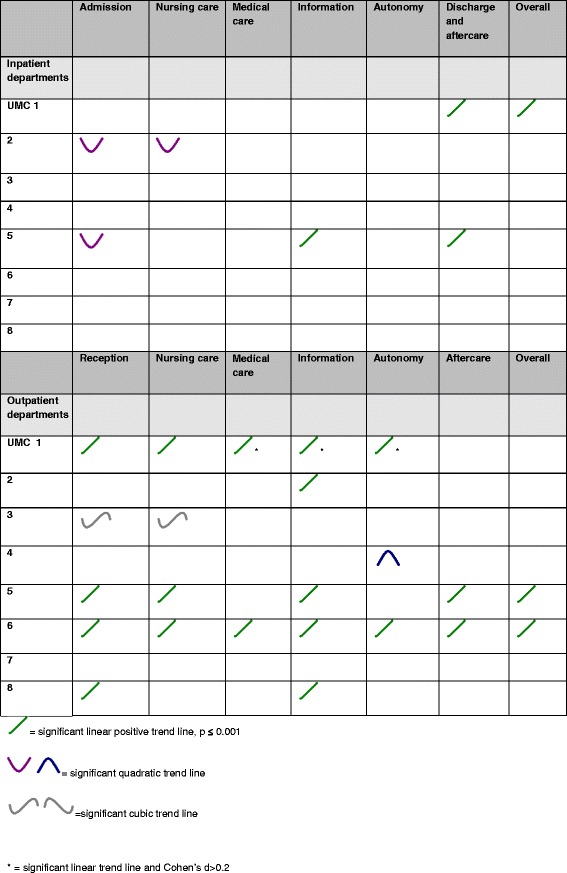
**Significant trends in patient satisfaction per dimension per university medical center.**

Figure 1 shows the significant trends in patient satisfaction per dimension for each university medical center. The statistics of the trends (means, SD, F-statistics and Cohen’s d) are found in the Additional file 1: Table S4 and S5.

On a hospital level patient satisfaction in the inpatient clinics of two out of the eight university medical centers showed a significant positive trend line on the dimension “Discharge and aftercare” and one on the dimension “Information”. UMC 2 showed two significant quadratic trend lines, on the dimensions “Admission” and “Nursing care”, UMC 5 showed a quadratic trend line on the dimension “Admission”. In both hospitals, satisfaction on these dimensions decreased in 2005 and remained stable in 2007, but showed an increase in 2009. The overall patient satisfaction score of university medical center 1 shows a significant positive amelioration.

In the outpatient clinics we found five university medical centers that showed significant positive trend lines on the dimension “Information”, four that showed a significant positive trend line on the dimension “Reception”. Three university medical centers show significant positive trend lines on “Nursing care” and two out of eight university medical centers showed positive trend lines on the dimensions “Medical care”, “Autonomy” and “Discharge and aftercare”. UMC 3 showed two significant cubic trend lines, on the dimensions “Reception” and “Nursing care”. Satisfaction on these dimensions decreased in 2005, but showed an increase in 2007, which was consolidated in 2009. UMC 4 showed a significant quadratic trend line on the dimension “Autonomy”: satisfaction on this dimension increased in 2005 and remained stable in 2007, but showed a decrease in 2009. The overall patient satisfaction scores of university medical center 5 and 6 show a significant positive increase.

University medical center 1 showed three significant (p ≤ 0,001) linear positive trend lines with an observed effect size expressed by Cohen’s d of 0.2: “Medical care” (Cohen’s d 0.20, 95% CI 0.14-0.26), “Information” (Cohen’s d 0.20, 95% CI 0.13-0.27) and “Autonomy” (Cohen’s d 0.20, 95% CI 0.14-0.27).

### Do hospitals with initial high satisfaction scores find room for improvement?

For inpatient departments, 26 percent of the patients is “very satisfied” on average (range 24 to 27 percent in 2003 and 22 to 27 percent in 2009). Table 1 shows the percentages very satisfied patients, their logit transformed scores and the change on both scores between 2003 and 2009 for inpatient departments on a hospital level (Table 1).

**Table 1:** **Percentage very satisfied patients, logit transformed score and change between 2003 and 2009 in inpatient departments**

**UMC**	**2003**		**2009**		**Change**	
	% very satisfied	Logit transformed score	% very satisfied	Logit transformed score	In % very satisfied	In logit transformed score
UMC 7	26.8	3.29	27.2	3.30	0.4	0.01
UMC 3	26.7	3.28	26.9	3.29	0.2	0.01
UMC 5	26.2	3.27	26.9	3.29	0.7	0.03
UMC 6	25.8	3.25	25.0	3.22	−0.8	−0.03
UMC 8	25.1	3.22	26.1	3.26	1.0	0.04
UMC 4	25.0	3.22	22.3	3.10	−2.7	−0.11
UMC 1	24.6	3.20	25.8	3.25	1.3	0.05
UMC 2	23.6	3.16	24.3	3.19	0.6	0.03

The university medical centers having the highest percentage very satisfied patients in 2003 (UMC 7 and 3) still showed an increase in very satisfied patients, although not as strongly as university medical centers with a lower initial proportion of very satisfied patients. Two average performing university medical centers show a decrease in satisfaction.

For the outpatient departments, 24 percent of the patients is “very satisfied” on average (range 21 to 25 percent in 2003 and 23 to 28 percent in 2009). Table 2 shows the percentages of very satisfied patients, their logit transformed scores and the change on both scores between 2003 and 2009 for outpatient departments on a hospital level (Table 2).

**Table 2:** **Percentage very satisfied patients, logit transformed score and change between 2003 and 2009 in outpatient departments**

**UMC**	**2003**		**2009**		**Change**	
	% very satisfied	Logit transformed score	% very satisfied	Logit transformed score	In % very satisfied	In logit transformed score
UMC 7	25	3.22	26.6	3.28	1.6	0.06
UMC 5	24.7	3.21	27.7	3.32	3	0.11
UMC 2	23.6	3.16	25.7	3.25	2.1	0.09
UMC 3	23.4	3.15	22.8	3.13	−0.6	−0.03
UMC 4	23.2	3.14	22.5	3.11	−0.7	−0.03
UMC 6	22.8	3.13	24.8	3.21	2	0.08
UMC 8	22.2	3.10	22.9	3.13	0.7	0.03
UMC 1	21.2	3.05	24.4	3.19	3.2	0.14

The highest increase in very satisfied patients was found in the university medical center with initially the lowest percentage very satisfied patients (UMC 1). The smallest change in very satisfied patients was found in UMC 3, 4 and 7, average to low performers. Two university medical centers performing on an average level, show a decrease in satisfaction. The university medical centers with the highest percentage of very satisfied patients (UMC 7, 5 and 2) still showed room for improvement.

## Discussion

Our study shows significant positive trend lines in patient satisfaction in the university medical centers in the period 2003–2009 on a national level. Also, several significant positive trend lines are found on a hospital level, especially in the outpatient departments. This is contrary to the fluctuating Dutch consumer confidence [34] and, according to the Dutch Health Care Consumer Panel, the declining confidence in Dutch hospitals: confidence decreased from 76% of the respondents in 2002 to 66% of the respondents in 2009 [35].

However, these increasing patient satisfaction scores are in line with the results from studies in Denmark, England, South Korea and the Netherlands [2,6,10,36-38]. An explanation for the increased patient satisfaction could be the quality interventions based on the results of the previous measurements. The dimensions “Information” and “Discharge and aftercare” showed most room for improvement in each measurement, with mean scores lower than the other dimensions. Indeed, most of the self-reported quality interventions made by the university medical centers concerned these dimensions. The results show significantly increased patient satisfaction on these dimensions in several university medical centers.

In comparing studies, the standardized effect size is a necessary statistic to provide some indication of practical significance of an effect [39,40]. To the best of our knowledge, however, this is the first study to report the magnitude of the change in patient satisfaction scores by means of Cohen’s d [28]. As a result, common effect sizes of patient satisfaction research are unknown. We know from literature that, when the sample size is large, the observed effect size is a good estimator of the true effect size [41]. However, there has always been discussion about the strong negative correlation between observed effect sizes and sample sizes [41,42]. As a result, large sample sizes studies like ours tend to report small or trivial effects. It is possible to obtain statistically significant results even for extremely small effects in large samples. On the other hand, large sample sizes give researchers the assurance of being capable of detecting an effect, even if it is small [41,42]. The observed effect size of the differences found, as referred to Cohen, is limited. However, it is not only the magnitude of effect that is important. Also, its practical value must be considered [29]. For instance, the fact that these four large-scale nationwide studies are comparable in instrument, methods, procedures and numbers of patients participating per hospital and specialty, gives weight to the relevance of the scores. Small effects may have enormous implications in a practical context, they may accumulate over time to become large effects and they may be quite important theoretically [40]. For instance, a small difference could nevertheless be important for patients. Whether the effects in patient satisfaction we observed are ‘practically relevant’ is hard to answer in the absence of other publications reporting effect sizes, and in the absence of other measures to compare the changes in patient satisfaction scores with.

According to the threshold of 15 percent applied in health status questionnaires [31,32], our study shows a ceiling effect: we found 24 percent of the outpatients and 26 percent of the patients in inpatient departments were “very satisfied”. However, the overall scores in our study are not as high as scores found in a study in inpatient departments in Norway [15]. Bjertnaes and colleagues found a mean satisfaction score of 4.2 on a 5-point Likert scale, where 5 represents the best score. Forty percent of the patients gave the highest score. In our study for inpatients the results were a mean of 3.9 and 26 percent respectively. Therefore, the decision to choose the intentionally skewed answering categories of the COPS in order to limit the ceiling effect of the questionnaire, seems to have worked out well [9,21].

High patient satisfaction scores do not mean there is no room for improvement [15,43]. In our data – even though changes are small -, a substantial number of scores is increasing over time, when controlled for a potential numerical ceiling effect, even in hospitals with initial high scores. Also, the biggest change is not found by definition in hospitals with a low initial score. We agree with Friesner and colleagues, who state that in patient satisfaction research the focus should be on maintaining the high mean scores while reducing the variation in responses [18]. Our data show that this was the case for almost all the university medical centers: the SD decreased in 2009 in inpatient and outpatient departments, with the exception of the SD of UMC 7 in inpatient and outpatient departments, and UMC 3 in inpatient departments (see Additional file 1: Table S6). Furthermore, the use of patient satisfaction research for quality improvement initiatives should preferably focus on the low scoring subgroups of patients while maintaining the high scores. Knowledge of the problems experienced within these subgroups of unsatisfied patients is valuable for tailoring quality improvement work [15,44] and may improve patient-centered care for all patients.

Finally, high patient satisfaction scores dropping down in time could indicate a stagnation of quality improvement. From this point of view monitoring patient satisfaction scores in time could be a useful indicator for the supervision and regulation of quality of care by, for instance, the national health care inspectorate.

### Strength and limitations

Given our large sample size, the uniform procedure followed by all eight university medical centers, we could well establish reliable changes in patient satisfaction over time. Another strength of our approach is that the quantitative results were supplemented by patient comments reported at department level, thus constituting a useful base for quality interventions [45]. Furthermore, the survey time of all university medical centers was equal and performed at the same time, thus there is no need to adjust for survey time in between-hospital comparisons [16,46].

However, our study also has some limitations. We did not perform a power calculation at the start of the measurements. Because of the significant differences found in our study, a post hoc power analysis is not of added value. In fact, the applications of post hoc power analyses are extremely limited [47]. It is for example not recommended because the observed power is almost always a biased estimator of the true power. If the goal is to determine the sample size needed to detect a practically important effect, one can refer to power tables mentioned by Cohen [27,41].

Also, the response rate dropped in 2009 from 53 to 42 per cent, while the satisfaction scores improved in 2009. Although the response rate was still reasonable [48], satisfied patients may have responded in particular. Given the anonymity of the study, no data to perform a nonresponse analysis were available. However, former research showed that the impact of nonresponse bias on satisfaction questionnaires of hospitalized patients is relatively small [9,49]. Furthermore, the satisfaction scores in case of significant trends not only improved in 2009, but also in 2005 and 2007.

Finally, the role of quality improvement activities by the hospitals is difficult to establish in this research. Although the results of the measurements gave direction to the topics of quality improvement activities the hospitals made, it is not possible to dissociate quality improvements from general organizational changes. Patient satisfaction scores may be difficult to interpret [2,14] and are influenced by many external factors. The environment in hospitals changes constantly. For instance, the economic situation [50], staff satisfaction [51], organizational circumstances [6,51] and public reporting of the results, especially for hospitals with low scores at the start are known factors to influence patient satisfaction results [6,8,14,19,52]. However, such external incentives alone are insufficient drivers of improvement efforts [11,53]. Enhancing internal motivation by integrating the cycle of measurement and improvements based on the results in the quality management system of the university medical centers almost certainly resulted in increased awareness about the patients’ perspective.

## Conclusions

We conclude that significant trends in patient satisfaction can be identified on a national and a hospital level, in inpatient and outpatient departments even though the observed effect size expressed by Cohen’s d is rather small. In other words, hospitals have found room for improvement, even when their scores were high initially. Our results indicate that monitoring patient satisfaction scores over time results may give valuable information on the effectiveness of quality programs for hospitals from patients’ perspective. Also, programs targeted at problem areas identified by patients might improve patient satisfaction scores. The quality interventions the hospitals made based on previous measurements and the increased awareness of the patients’ perspective could be possible mechanisms leading to the increased patient satisfaction scores. However, to complete the picture, future patient satisfaction research should keep track of external and organizational circumstances that might influence these scores. In the original measurement reports sent to the university medical centers results were presented on department level, so quality interventions could be tailored to department-specific targets. It would be valuable to also expand future research to identify subgroups of unsatisfied patients. In this way a twofold value is added: unsatisfied patients can indicate risks in quality or safety aspects in hospital care, and tailoring quality interventions to this subgroup can lead to improved patient-centered care for all patients.

## Additional file

Additional file 1: Table S1. Number of patients in de datasets, per year, hospital and total. **Table S2.** Patient characteristics inpatient departments (%). **Table S3.** Patient characteristic outpatient departments (%). **Table S4.** Trend statistics for significant trend lines per university medical centre for inpatient departments (trend, means, SD, absolute mean difference, Cohen’s d (95% CI), F-statistics, p-value, N). **Table S5.** Trend statistics for significant trend lines per university medical centre for outpatient departments (trend, means, SD, absolute mean difference, Cohen’s d (95% CI), F-statistics, p-value, N). **Table S6.** Overall UMC SD-scores in time.
