# Supplementary material for: Trends in patient satisfaction in Dutch university medical centers: room for improvement for all
Source: BMC Health Serv Res. 2015 Mar 19;15:112. doi: 10.1186/s12913-015-0766-7 (PMC4404205; doi:10.1186/s12913-015-0766-7)
Supplement: Additional file 1: Table S1. — Number of patients in de datasets, per year, hospital and total. Table S2. Patient characteristics inpatient departments (%). Table S3. Patient characteristic outpatient departments (%). Table S4. Trend statistics for significant trend lines per university medical centre for inpatient departments (trend, means, SD, absolute mean difference, Cohen’s d (95% CI), F-statistics, p-value, N). Table S5. Trend statistics for significant trend lines per university medical centre for outpatient departments (trend, means, SD, absolute mean difference, Cohen’s d (95% CI), F-statistics, p-value, N). Table S6. Overall UMC SD-scores in time. [file 12913_2015_766_MOESM1_ESM.doc]

Appendix

Table 1 Number of patients in de datasets, per year, hospital and total

|  | Inpatient | | | | | Outpatient | | | | |
| --- | --- | --- | --- | --- | --- | --- | --- | --- | --- | --- |
|  | 2003 | 2005 | 2007 | 2009 | Total | 2003 | 2005 | 2007 | 2009 | Total |
| UMC 1 | 2,206 | 1,943 | 1,863 | 1,605 | 7,617 | 2,167 | 2,428 | 2,441 | 2,325 | 9,361 |
| 2 | 1,483 | 1,399 | 1,654 | 1,340 | 5,876 | 2,156 | 1,820 | 2,093 | 1,869 | 7,938 |
| 3 | 1,978 | 2,040 | 1,564 | 1,704 | 7,286 | 2,714 | 2,687 | 2,745 | 2,962 | 11,108 |
| 4 | 1,611 | 1,627 | 1,301 | 1,806 | 6,345 | 2,175 | 2,237 | 2,850 | 1,876 | 9,138 |
| 5 | 1,800 | 1,818 | 2,095 | 1,677 | 7,390 | 2,565 | 2,320 | 3,185 | 3,151 | 11,221 |
| 6 | 2,470 | 1,994 | 1,910 | 1,758 | 8,132 | 2,651 | 2,539 | 2,707 | 2,134 | 10,031 |
| 7 | 2,290 | 2,245 | 2,234 | 1,819 | 8,588 | 2,870 | 2,637 | 2,375 | 2,192 | 10,074 |
| 8 | 1,532 | 1,960 | 1,956 | 1,373 | 6,821 | 3,816 | 2,420 | 2,653 | 1,738 | 10,627 |
| Total | 15,370 | 15,026 | 14,577 | 13,082 | 58,055 | 21,114 | 19,088 | 21,049 | 18,247 | 79,498 |

Table 2 Patient characteristics inpatient departments (%)

|  |  | 2003 | 2005 | 2007 | 2009 |
| --- | --- | --- | --- | --- | --- |
| Gender | Male | 50.3 | 49.0 | 48.6 | 48.5 |
|  | Female | 49.7 | 51.0 | 51.4 | 51.5 |
| Education* | None | 15.1 | 14.1 | 13.2 | 12.2 |
|  | Lower education | 26.1 | 25.5 | 24.2 | 22.9 |
|  | Middle education | 29.6 | 30.6 | 30.9 | 31.7 |
|  | Higher education | 8.7 | 8.7 | 8.8 | 8.5 |
|  | College | 14.5 | 14.7 | 15.7 | 17.4 |
|  | University | 6.1 | 6.4 | 7.2 | 7.3 |
| Age* | Younger than 20 years | 14.7 | 13.7 | 12.4 | 11.0 |
|  | 20-39 years | 17.7 | 16.8 | 16.0 | 16.1 |
|  | 40-54 years | 20.4 | 19.4 | 19.0 | 18.9 |
|  | 55-59 years | 10.0 | 9.8 | 9.9 | 9.5 |
|  | 60 years and older | 37.3 | 40.3 | 42.7 | 44.5 |
| Health status* | Bad | 5.6 | 5.3 | 5.8 | 4.7 |
|  | Moderate | 27.1 | 27.3 | 27.5 | 26.5 |
|  | Good | 45.3 | 45.2 | 45.1 | 46.6 |
|  | Very good | 13.9 | 14.2 | 14.3 | 15.1 |
|  | Excellent | 8.0 | 8.0 | 7.3 | 7.2 |

* Pearsons chi-square < 0.05

Table 3 Patient characteristic outpatient departments (%)

|  |  | 2003 | 2005 | 2007 | 2009 |
| --- | --- | --- | --- | --- | --- |
| Gender | Male | 43.2 | 43.9 | 44.3 | 43.7 |
|  | Female | 56.8 | 56.1 | 55.7 | 56.3 |
| Education* | None | 13.9 | 11.7 | 11.8 | 9.3 |
|  | Lower education | 23.5 | 22.5 | 21.6 | 21.0 |
|  | Middle education | 29.4 | 30.5 | 30.2 | 32.1 |
|  | Higher education | 10.1 | 9.7 | 10.1 | 9.7 |
|  | College | 15.6 | 17.2 | 17.5 | 18.7 |
|  | University | 7.5 | 8.3 | 8.8 | 9.2 |
| Age* | Younger than 20 years | 14.7 | 12.2 | 11.4 | 9.2 |
|  | 20-39 years | 19.7 | 19.3 | 17.3 | 16.8 |
|  | 40-54 years | 22.0 | 22.7 | 21.8 | 20.9 |
|  | 55-59 years | 9.9 | 10.7 | 10.3 | 10.1 |
|  | 60 years and older | 33.7 | 35.2 | 39.2 | 43.0 |
| Health status* | Bad | 5.3 | 5.0 | 4.8 | 4.4 |
|  | Moderate | 31.7 | 29.4 | 30.8 | 30.8 |
|  | Good | 45.4 | 46.8 | 46.5 | 47.0 |
|  | Very good | 12.0 | 12.6 | 12.6 | 12.9 |
|  | Excellent | 5.5 | 6.1 | 5.3 | 4.9 |

* Pearsons chi-square < 0.05

Table 4 Trend statistics for significant trend lines per university medical centre for inpatient departments (trend, means, SD, absolute mean difference, Cohen’s d (95% CI), F-statistics, p-value, N)

|  | **Trend** | **Mean** | | **Absolute mean difference (2003-2009)** | **SD** | **Cohen’s d (95% CI)** | **F (df)** | **p-value** | **N** |
| --- | --- | --- | --- | --- | --- | --- | --- | --- | --- |
| UMC 1 Discharge and aftercare | 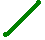 | 2003 | 3.42 | 0.17 | 1.03 | 0.16  (0.08-  0.24) | 6.322 (3) | <0.001 | 4,983 |
|  |  | 2005 | 3.47 |  | 0.99 |  |  |  |  |
|  |  | 2007 | 3.52 |  | 0.99 |  |  |  |  |
|  |  | 2009 | 3.58 |  | 0.99 |  |  |  |  |
| UMC 1 Overall | 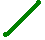 | 2003 | 3,75 | 0.11 | 0,75 | 0.16  (0.08-0.25) | 5.443 (3) | 0.001 | 4,348 |
|  |  | 2005 | 3,78 |  | 0,72 |  |  |  |  |
|  |  | 2007 | 3,84 |  | 0,73 |  |  |  |  |
|  |  | 2009 | 3,87 |  | 0,74 |  |  |  |  |
| UMC 2 Admission | 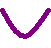 | 2003 | 3.94 | 0.03 | 0.80 | - | 8.107 (3) | <0.001 | 5,139 |
|  |  | 2005 | 3.83 |  | 0.81 |  |  |  |  |
|  |  | 2007 | 3.86 |  | 0.83 |  |  |  |  |
|  |  | 2009 | 3.97 |  | 0.80 |  |  |  |  |
| UMC 2  Nursing care | 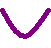 | 2003 | 3.95 | 0.01 | 0.87 | - | 8.428 (3) | <0.001 | 5,336 |
|  |  | 2005 | 3.83 |  | 0.88 |  |  |  |  |
|  |  | 2007 | 3.83 |  | 0.90 |  |  |  |  |
|  |  | 2009 | 3.96 |  | 0.88 |  |  |  |  |
| UMC 5 Admission | 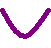 | 2003 | 3.96 | 0.07 | 0.83 | - | 6,13 (3) | <0.001 | 6,415 |
|  |  | 2005 | 3.93 |  | 0.80 |  |  |  |  |
|  |  | 2007 | 3.92 |  | 0.86 |  |  |  |  |
|  |  | 2009 | 4.03 |  | 0.83 |  |  |  |  |
| UMC 5 Information | 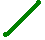 | 2003 | 3.66 | 0.11 | 0.88 | 0.11  (0.08-0.19) | 6,142 (3) | <0.001 | 6,508 |
|  |  | 2005 | 3.64 |  | 0.85 |  |  |  |  |
|  |  | 2007 | 3.72 |  | 0.84 |  |  |  |  |
|  |  | 2009 | 3.76 |  | 0.87 |  |  |  |  |
| UMC 5 Discharge and aftercare | 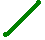 | 2003 | 3,52 | 0.16 | 1,02 | 0.16  (0.08-0.24) | 9.397 (3) | <0.001 | 4,872 |
|  |  | 2005 | 3,47 |  | 0,98 |  |  |  |  |
|  |  | 2007 | 3,55 |  | 0,98 |  |  |  |  |
|  |  | 2009 | 3,68 |  | 0,97 |  |  |  |  |


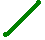
= significant linear positive trend line, p ≤ 0.001


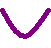
 = significant quadratic trend line

Table 5 Trend statistics for significant trend lines per university medical centre for outpatient departments (trend, means, SD, absolute mean difference, Cohen’s d (95% CI), F-statistics, p-value, N)

|  | **Trend** | **Mean** | | **Absolute mean difference (2003-2009)** | **SD** | **Cohen’s d (95% CI))** | **F (df)** | **p-value** | **N** |
| --- | --- | --- | --- | --- | --- | --- | --- | --- | --- |
| UMC 1 Reception | 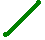 | 2003 | 3.75 | 0.16 | 0.81 | 0,19  (0.13 -0.25) | 12.867 (3) | <0.001 | 8,648 |
|  |  | 2005 | 3.80 |  | 0.78 |  |  |  |  |
|  |  | 2007 | 3.87 |  | 0.77 |  |  |  |  |
|  |  | 2009 | 3.90 |  | 0.76 |  |  |  |  |
| UMC 1 Nursing care | 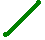 | 2003 | 3.77 | 0.13 | 0.87 | 0.15  (0.09-0.22) | 7.002 (3) | <0.001 | 8,661 |
|  |  | 2005 | 3.82 |  | 0.84 |  |  |  |  |
|  |  | 2007 | 3.88 |  | 0.81 |  |  |  |  |
|  |  | 2009 | 3.90 |  | 0.81 |  |  |  |  |
| UMC 1 Medical care | 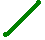* | 2003 | 4.00 | 0.19 | 0.96 | **0.20**  **(0.14-0.26)** | 11.746 (3) | <0.001 | 8,674 |
|  |  | 2005 | 4.10 |  | 0.92 |  |  |  |  |
|  |  | 2007 | 4.10 |  | 0.90 |  |  |  |  |
|  |  | 2009 | 4.18 |  | 0.86 |  |  |  |  |
| UMC 1 Information | 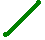* | 2003 | 3.61 | 0.16 | 0.90 | **0.20**  **(0.13-0.27)** | 8.143 (3) | <0.001 | 6,831 |
|  |  | 2005 | 3.66 |  | 0.87 |  |  |  |  |
|  |  | 2007 | 3.70 |  | 0.86 |  |  |  |  |
|  |  | 2009 | 3.78 |  | 0.84 |  |  |  |  |
| UMC 1 Autonomy | 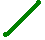* | 2003 | 3.70 | 0.16 | 0.83 | **0.20**  **(0.14-0.27)** | 12.825 (3) | <0.001 | 8,505 |
|  |  | 2005 | 3.82 |  | 0.79 |  |  |  |  |
|  |  | 2007 | 3.79 |  | 0.79 |  |  |  |  |
|  |  | 2009 | 3.86 |  | 0.74 |  |  |  |  |
| UMC 2 Information | 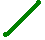 | 2003 | 3.64 | 0.16 | 0.92 | 0.18  (0.11-0.25) | 5.229 (3) | 0.001 | 5,636 |
|  |  | 2005 | 3.70 |  | 0.86 |  |  |  |  |
|  |  | 2007 | 3.75 |  | 0.84 |  |  |  |  |
|  |  | 2009 | 3.80 |  | 0.85 |  |  |  |  |
| UMC 3 Reception | 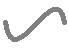 | 2003 | 3.84 | 0.07 | 0.75 | - | 12.229 (3) | <0.001 | 10,242 |
|  |  | 2005 | 3.81 |  | 0.77 |  |  |  |  |
|  |  | 2007 | 3.92 |  | 0.74 |  |  |  |  |
|  |  | 2009 | 3.91 |  | 0.75 |  |  |  |  |
| UMC 3 Nursing care | 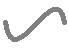 | 2003 | 3.86 | 0.06 | 0.80 | - | 11.835 (3) | <0.001 | 10,252 |
|  |  | 2005 | 3.83 |  | 0.83 |  |  |  |  |
|  |  | 2007 | 3.94 |  | 0.78 |  |  |  |  |
|  |  | 2009 | 3.92 |  | 0.78 |  |  |  |  |
| UMC 4 Autonomy | 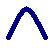 | 2003 | 3.70 | 0.01 | 0.86 | - | 6.183 (3) | <0.001 | 8,268 |
|  |  | 2005 | 3.79 |  | 0.82 |  |  |  |  |
|  |  | 2007 | 3.78 |  | 0.79 |  |  |  |  |
|  |  | 2009 | 3.71 |  | 0.81 |  |  |  |  |
| UMC 5 Reception | 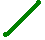 | 2003 | 3.93 | 0.11 | 0.73 | 0.16  (0.10-0.21) | 7.083 (3) | <0.001 | 10,349 |
|  |  | 2005 | 3.95 |  | 0.72 |  |  |  |  |
|  |  | 2007 | 3.98 |  | 0.72 |  |  |  |  |
|  |  | 2009 | 4.04 |  | 0.69 |  |  |  |  |
| UMC 5 Nursing care | 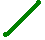 | 2003 | 3.95 | 0.10 | 0.78 | 0.15  (0.09-0.20) | 8.512 (3) | <0.001 | 10,376 |
|  |  | 2005 | 3.95 |  | 0.77 |  |  |  |  |
|  |  | 2007 | 4.01 |  | 0.73 |  |  |  |  |
|  |  | 2009 | 4.06 |  | 0.73 |  |  |  |  |
| UMC 5 Information | 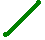 | 2003 | 3.71 | 0.16 | 0.86 | 0.19  (0.13-0.25) | 8.942 (3) | <0.001 | 8,271 |
|  |  | 2005 | 3.76 |  | 0.85 |  |  |  |  |
|  |  | 2007 | 3.78 |  | 0.84 |  |  |  |  |
|  |  | 2009 | 3.87 |  | 0.83 |  |  |  |  |
| UMC 5 Aftercare | 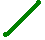 | 2003 | 3.38 | 0.16 | 1.01 | 0.17  (0.11-0.24) | 5.311 (3) | 0.001 | 6,771 |
|  |  | 2005 | 3.47 |  | 0.98 |  |  |  |  |
|  |  | 2007 | 3.46 |  | 0.97 |  |  |  |  |
|  |  | 2009 | 3.55 |  | 0.96 |  |  |  |  |
| UCM 5 Overall | 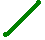 | 2003 | 3,85 | 0.12 | 0,70 | 0.18  (0.10-0.25) | 5.237 (3) | 0.001 | 5,629 |
|  |  | 2005 | 3,89 |  | 0,69 |  |  |  |  |
|  |  | 2007 | 3,89 |  | 0,69 |  |  |  |  |
|  |  | 2009 | 3,97 |  | 0,66 |  |  |  |  |
| UMC 6 Reception | 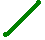 | 2003 | 3.92 | 0.10 | 0.72 | 0.13  (0.07-0.19) | 10.038 (3) | <0.001 | 9,334 |
|  |  | 2005 | 3.92 |  | 0.73 |  |  |  |  |
|  |  | 2007 | 3.99 |  | 0.70 |  |  |  |  |
|  |  | 2009 | 4.01 |  | 0.69 |  |  |  |  |
| UMC 6 Nursing care | 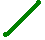 | 2003 | 3.90 | 0.09 | 0.77 | 0.12  (0.0.6-0.18) | 8.611 (3) | <0.001 | 9,331 |
|  |  | 2005 | 3.91 |  | 0.78 |  |  |  |  |
|  |  | 2007 | 3.99 |  | 0.75 |  |  |  |  |
|  |  | 2009 | 3.99 |  | 0.74 |  |  |  |  |
| UMC 6 Medical care | 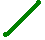 | 2003 | 4.02 | 0.14 | 0.94 | 0.14  (0.09-0.20) | 14.475 (3) | <0.001 | 9,336 |
|  |  | 2005 | 4.02 |  | 0.93 |  |  |  |  |
|  |  | 2007 | 4.14 |  | 0.87 |  |  |  |  |
|  |  | 2009 | 4.15 |  | 0.85 |  |  |  |  |
| UMC 6 Information | 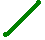 | 2003 | 3.63 | 0.17 | 0.90 | 0.19  (0.13-0.26) | 14.539 (3) | <0.001 | 7,512 |
|  |  | 2005 | 3.66 |  | 0.90 |  |  |  |  |
|  |  | 2007 | 3.78 |  | 0.82 |  |  |  |  |
|  |  | 2009 | 3.80 |  | 0.86 |  |  |  |  |
| UMC 6 Autonomy | 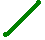 | 2003 | 3.80 | 0.09 | 0.78 | 0.12  (0.06-0.18) | 5.319 (3) | 0.001 | 9,157 |
|  |  | 2005 | 3.84 |  | 0.79 |  |  |  |  |
|  |  | 2007 | 3.88 |  | 0.76 |  |  |  |  |
|  |  | 2009 | 3.89 |  | 0.74 |  |  |  |  |
| UMC 6 Aftercare | 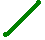 | 2003 | 3.37 | 0.14 | 1.01 | 0.14  (0.07-0.21) | 7.327 (3) | <0.001 | 6,353 |
|  |  | 2005 | 3.45 |  | 0.98 |  |  |  |  |
|  |  | 2007 | 3.53 |  | 0.97 |  |  |  |  |
|  |  | 2009 | 3.51 |  | 0.99 |  |  |  |  |
| UMC 6 Overall | 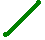 | 2003 | 3,79 | 0.12 | 0,72 | 0.17  (0.09-0.25) | 9,956 (3) | <0.001 | 5,385 |
|  |  | 2005 | 3,83 |  | 0,71 |  |  |  |  |
|  |  | 2007 | 3,93 |  | 0,66 |  |  |  |  |
|  |  | 2009 | 3,91 |  | 0,68 |  |  |  |  |
| UMC 8 Reception | 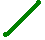 | 2003 | 3.75 | 0.12 | 0.78 | 0.16  (0.10-0.21) | 6,501 (3) | <0.001 | 9,779 |
|  |  | 2005 | 3.76 |  | 0.77 |  |  |  |  |
|  |  | 2007 | 3.82 |  | 0.77 |  |  |  |  |
|  |  | 2009 | 3.87 |  | 0.76 |  |  |  |  |
| UMC 8 Information | 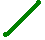 | 2003 | 3.64 | 0.11 | 0.87 | 0.13  (0.06-0.19) | 6.136 (3) | <0.001 | 7,567 |
|  |  | 2005 | 3.61 |  | 0.89 |  |  |  |  |
|  |  | 2007 | 3.71 |  | 0.84 |  |  |  |  |
|  |  | 2009 | 3.75 |  | 0.84 |  |  |  |  |


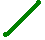
= significant linear positive trend line, p ≤ 0.001


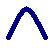
= significant quadratic trend line


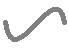
 =significant cubic trend line

* = significant linear trend line and Cohen’s d>0.2

Table 6 Overall UMC SD-scores in time

|  |  | Inpatient | Outpatient |
| --- | --- | --- | --- |
| UMC 1 | 2003 | 0.75 | 0.76 |
|  | 2009 | 0.74 | 0.70 |
| UMC 2 | 2003 | 0.74 | 0.74 |
|  | 2009 | 0.69 | 0.70 |
| UMC 3 | 2003 | 0.69 | 0.73 |
|  | 2009 | 0.70 | 0.68 |
| UMC 4 | 2003 | 0.78 | 0.73 |
|  | 2009 | 0.74 | 0.73 |
| UMC 5 | 2003 | 0.72 | 0.70 |
|  | 2009 | 0.71 | 0.66 |
| UMC 6 | 2003 | 0.68 | 0.72 |
|  | 2009 | 0.68 | 0.68 |
| UMC 7 | 2003 | 0.69 | 0.68 |
|  | 2009 | 0.70 | 0.71 |
| UMC 8 | 2003 | 0.74 | 0.71 |
|  | 2009 | 0.72 | 0.70 |
